# Building a Digital Wind Farm

**DOI:** 10.1007/s11831-017-9222-7

**Published:** 2017-04-18

**Authors:** Sam Hewitt, Lee Margetts, Alistair Revell

**Affiliations:** School of Mechanical, Aerospace and Civil Engineering, The University of Manchester, Manchester, M13 9PL UK

**Keywords:** Wind energy, Simulation, Modelling, Aerodynamics, Fluid–structure interaction

## Abstract

The purpose of this paper is to provide a high level, holistic overview of the work being undertaken in the wind energy industry. It summarises the main techniques used to simulate both aerodynamic and structural issues associated with wind turbines and farms. The motivation behind this paper is to provide new researchers with an outlook of the modelling and simulation landscape, whilst highlighting the trends and direction research is taking. Each section summarises an individual area of simulation and modelling, covering the important historical research findings and a comprehensive analysis of recent work. This segregated approach emphasises the key components of wind energy. Topics range in geometric scales and detail, ranging from atmospheric boundary layer modelling, to fatigue and fracture in the turbine blades. More recent studies have begun to combine a range of scales and physics to better approximate real systems and provide higher fidelity and accurate analyses to manufacturers and companies. This paper shows a clear trend towards coupling both scales and physics into singular models utilising high performance computing system.

## Introduction

With an ever growing population size and the improvement in living standards, the demand and consumption of energy is only going to escalate. Considering the surge in power consumption in combination with growing concerns about the finite nature of fossil fuels and the impact they can have on the climate, there has been a concerted effort from governments to encourage development and integration of more renewable sources of energy. This push has come through social and economic policies, such as the EU 2030 climate and energy framework [1] and the US “Clean Power Plan” [2]. Along with the obvious benefits it can have for the climate, a diverse set of energy sources can provide a greater level of economic and social security against possible disruptions [3].

In 2015, renewable sources of energy covered approximately $23\%$ of the UK’s electricity consumption and $11\%$ of the US energy production [4]. In the UK, wind represented $67\%$ of these sources [5], however there still requires a significant level of installation, of both onshore and offshore wind farms, to begin playing a significant role in meeting growing energy demands. The levelised cost (lifetime cost of the system divided by its total energy output) of wind power for projects starting in 2019 currently stands at approximately 99 and 107 £$/{\mathrm{MWh}}$ for onshore and offshore projects respectively. These costs are currently only surpassed by technologies such as nuclear, 80 £$/{\mathrm{MWh}}$ and combined coal and gas turbines, 85 £$/{\mathrm{MWh}}$ [6].

Technological advances in material science have allowed turbine manufacturers to build taller turbines with larger rotor diameters with a minimal increase in weight and cost. These turbines are then able to cover and capture more of the energy at higher altitudes where the wind is faster and more stable over a 24 h period. As the size of turbines grows, the blades become more flexible and it becomes increasingly important to simulate and understand both the complex flow physics and structural performance of these systems.

**Fig. 1:**
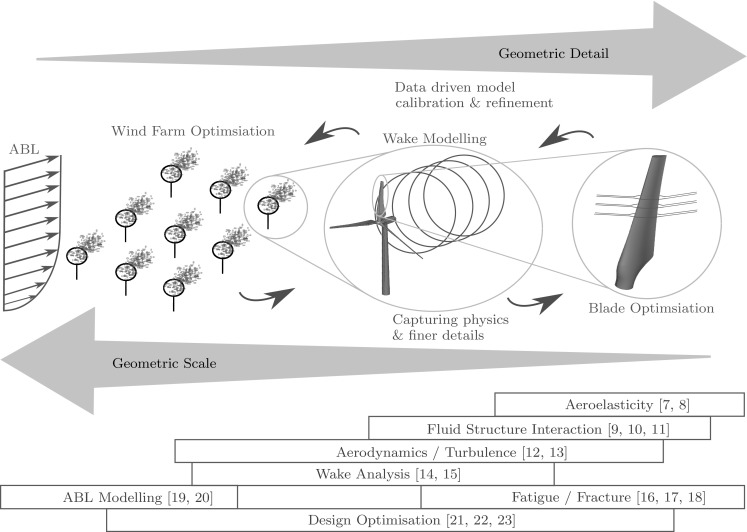
Geometric scale and detail of the major research areas in the wind industry

Figure 1 shows some of the key areas involved in simulating wind turbines. This review covers the major research being done within the aerodynamic and mechanical areas, shown in red and blue. It is composed of the following parts. Section 2 summarises the previous reviews concerned with wind energy and underlines the novelty and importance of the current paper. The following sections begin to cover the areas of research being undertaken in the industry. Section 3 considers the wind as a resource and some of the concepts involved, whilst Sects. 4 and 5 describe the major rotor models developed and the studies being undertaken into turbine arrays. Section 6 describes the work being done in turbine mechanics from blade failure to structural optimisation techniques. Finally Sect. 7 describes the most recent studies, combining the state of the art in structural mechanics methods with CFD simulations (fluid–structure interaction) before a conclusion summarising the general trends in the industry.

## Previous Literature Reviews

In 1998 Snel [24] produced one of the first reviews considering the simulation methodologies used to study aero elasticity in wind turbine rotors. This was soon followed by two papers in 2000 and 2002 by Akermann and Soder [25, 26], describing the history of wind turbines and the basics behind the technology. This included simple analytical approaches to modelling the wind, how turbines and turbine arrays are integrated into the network, some of the economics and siting issues and some special applications for wind turbines. Sahin [27] then provided a much more in depth review of wind as a resource and the economic factors involved in wind energy, before concluding with a current state of the market across the continents. In 2007 Joselin Herbert [28] provided a summary of wind assessment techniques and site selection methodologies, along with some general issues with components present in the turbine nacelle, such as the gearbox and generator.

A number of authors have focused on the environmental impacts of harvesting wind energy [29–32]. Pryor et al. [29] analysed the effects that the changing climate can have on wind turbine technology, whilst Leung and Yang [31] and Dai et al. [32] considered the environmental effects wind turbines cause, such as noise and visual pollution as well as some of the effects they can have on animals and radio systems.

Aero elasticity and the aerodynamics of wind turbines was covered by Hansen et al. [7] in 2006, discussing structural and aerodynamic modelling of wind turbines. It covered recent models used to represent the rotor and wake aerodynamics, along with simple structural models currently used to model rotor instabilities. Then in 2011 Zhang and Huang [8] provided a more up to date collection of the work done in aero elasticity and some areas they believed should be investigated in the future. Also in 2011 Sanderse et al. [14] provided a good summary of work undertaken in the field of wake analysis using more recent CFD models developed.

In 2010 Barlas and Van Kuik [33] reported on the state of the art in rotor control. The review analysed advanced control techniques and their use for load reduction in wind turbines. Four years later in 2014, Cheng et al. [34] explored the state of the art in generators and control methods, studying the latest technologies for wind energy conversion systems, along with the general trends and future possibilities of generation modules. In 2015 Hossain and Ali [35] discussed the future direction of generation mechanisms in turbines and how they would deal with the intermittent nature of wind. In the same year Rahman et al. [36] reviewed the control methods in wind turbines, considering both active and passive control methods used to limit vibrations and noise in the structure and blades.

The use of vertical axis turbines in an offshore application has been reviewed by Borg et al. in a number of papers [37–39], which cover the aerodynamic models used, the methods of mooring the turbines and mathematical models to simulate the combination of wind and wave induced oscillations. In 2015 Benitz et al. [40] covered the hydrodynamics of offshore applications, including water wave theory, ocean physics and how these effect structures such as turbines moored at sea.

Recently, Chehouri et al. [21] reviewed the optimisation methods used to design structural components and blades in wind turbines, covering the optimisation functions and algorithms used to explore the design space. McKenna et al. [41] have described the key challenges present in the wind industry and the future possibilities. The authors concluded that one of the key challenges was the structural loading in the blades and proposed that better modelling techniques to simulate the complex deformation could provide a solution.

**Table 1:** Summary of previous literature reviews

Topic	Papers	Year
Aerodynamics	[7, 8, 14]	2006, 2011, 2011
Wind resource	[28]	2007
Environment	[30–32]	2011, 2012, 2015
Optimisation	[21]	2015
Offshore	[37–39]	2014, 2014, 2015
Generation	[35, 36]	2015, 2015

Table 1 summarises the papers mentioned in this section into specific topics. This review provides a state of the art summary of simulation methodologies used in the wind energy industry. Providing this high level summary begins to highlight the implications and relevance of each individual subcomponent of “Wind Energy” and how these components aid the design and manufacturing process in the industry.

It looks at the development of the models used to simulate both aerodynamic and structural features within the wind turbine. A review of the recent trends in wind resource and atmospheric modelling on a number of length and time scales is provided which, to the authors knowledge, has not been reported on previously.

## Wind Resource Modelling

In the 1980s, as wind turbine technology was becoming more utilised, the ability to reliably and accurately predict weather conditions became more important. This in turn motivated a need to develop more accurate models and methods to predict wind speed patterns. Research led to the developments by the National Renewable Energy Laboratory (NREL), in the US, of the Wind Energy Resource Atlas of the United States [42] and the European Wind Atlas by The Technical University of Denmark [43] which describe the wind profiles over both temporal and spatial scales and are used in the initial stages of the siting process for wind farms.

The earth’s atmosphere is split up into layers, however the maximum height of a wind turbine currently stands at approximately 220 m, it is therefore only the lower portion of the atmosphere that concerns the wind industry. This lower layer is known as the troposphere, reaching approximately 12 km from the earth’s surface. This is subsequently split up into smaller regions, the Atmospheric Boundary Layer (ABL) and the free atmosphere [44].

### ABL Modelling

Early work on ABL modelling began in the 1970s. From the 70s to late 90s the work undertaken considered 2D flow in complex terrain [45–50]. These ranged from analytical approximations as in [48] to numerical simulations considering single, shallow hills [45, 46] and multiple hills ranging in steepness [51, 52], along with different approaches to modelling [49, 50], using a stream-function/vorticity numerical scheme, and parameter space studies [53, 54] looking at appropriate boundary conditions to generate accurate results.

In parallel to the work described above, researchers began considering Large Eddy Simulations (LES) and Reynolds Averaged Navier–Stokes (RANS) models to improve the understanding of the physics in the ABL. Deardorff [55] was one of the first to use LES in considering the stability of the ABL due to heat fluxes. Following on from the work done by Deardorff, in 1984 Moeng [56] developed a new LES model using a pseudo spectral representation of properties in the horizontal directions. Raithby [57] tested a new second order closure model using RANS on Askervein hill, an isolated area, for which experimental data was available.

At the turn of the 21st century, with increased computational power and better solution algorithms, there was an increase in the number of studies considering more complex physics involving ‘LES’ or ‘RANS’ and ‘Boundary Layer’ [58].

Extending the work done by Richards and Hoxey [54] in 1993, a number of authors concentrated on the development and validation of more accurate turbulence models and inflow parameters used in RANS simulations for flows over complex terrains [59–66]. Hargreaves et al. [61, 62] concentrated on the implementation of a new *k*−$\epsilon$ turbulence model in the commercial codes CFX and Fluent to maintain a more consistent ABL, which they suggested decayed too quickly using some of the previously developed models. Many of the studies concerned with ABL modelling [20, 60, 65, 67] used Askervein hill as a test case.

LES also played a significant role in the development of ABL modelling in the early 2000s. After initial studies in the late 1980s and 1990s considering LES [56, 68–70], a wide range of work has been done in improving the sub grid scale models of turbulence [67, 71–73]. In particular Bechmann began developing a *k*−$\epsilon$ LES model during his PhD in 2006 [67] and subsequently developed a Hybrid RANS/LES model [20] in order to combine the added physics of the LES with the more relaxed mesh requirements near the wall/boundary of RANS. In 2011 he used the Bolund experiment to compare the results from RANS and LES simulations [74]. The Bolund experiment was developed to provide a validation case for CFD codes studying complex terrains. Extensive measurements were taken over the hill, in Denmark, which induces complex 3D flows. It was found that RANS simulations with a two equation turbulence model offered better accuracy than the LES simulations, however mean errors for speed up of flow over the hill and turbulent kinetic energy were still quite high, 10 and 22% respectively.

More recently in 2012 and 2016 authors Balough [19] and Yan [75] have continued to develop and work on the *k*−$\epsilon$ turbulence model and implementation of boundary conditions for improved ABL modelling in complex terrains.

**Fig. 2:**
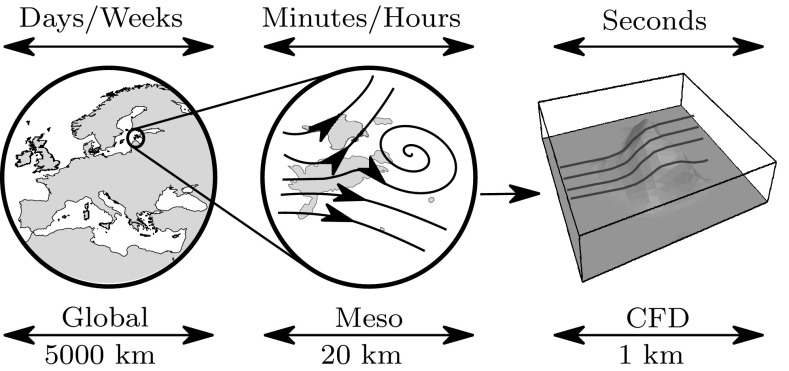
Time and space scales of the flow dynamics within the atmosphere

Work is being done by a number of authors [76, 77] into coupling the different scales of flow seen in the atmosphere. Mesoscale atmospheric models that capture effects down to around 1 km are being coupled to CFD simulations, particularly LES models that can simulate the smaller detail down to approximately 50 m, see Fig. 2. CFD studies struggle to incorporate all the atmospheric phenomena that mesoscale models such as the Weather Research and Forecasting Model (WRF) can, but are able to model more local effects such as hills and turbine wakes. This coupling ultimately improves the fidelity of simulations, by providing more realistic input profiles and improving the resource assessment process.

The work in coupling scales of flow is providing more realistic input profiles however, the state of the art in ABL modelling over complex terrains seems to be encased in RANS simulations with two equation closure models. Although LES can provide a more complete analysis of turbulent properties, until the accuracy and time limitations can be improved, RANS simulations will be used by both researchers and industry in modelling flows in complex terrains.

The studies described above have built a foundation for researchers interested in wind turbines. Models have begun to combine both the ABL modelling along with the interesting flow physics generated by turbines and farms. These will be described in Sect. 5.

## Rotor Modelling Methods

**Fig. 3:**
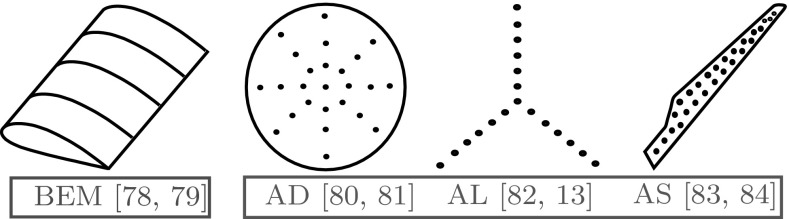
Simplified structural and CFD models of turbine rotors

Modelling wind turbine rotors can range in the level of sophistication and time requirements. Figure 3 shows some of the simplified structural and CFD models used in a large number of studies.

Some of the simpler models are usually used for larger computations that are concerned with the effect wind farms can have on the ABL [85–87] and how wakes interact [88–90]. More detailed analysis requires fully resolved geometric models [91–93] that provide much more information about the loads on the turbine rotor and structure, along with better predictions about power production.

### Blade Element Momentum Theory (BEM)

The most simple method used to predict blade loads is BEM, which is based on simple assumptions including analysing the force on the blade through control volumes and the conservation of linear and angular momentum. For a full derivation see [94].

BEM theory can provide quick, computationally cheap and reasonably accurate results [95] for steady conditions with a wake in equilibrium. The original BEM has been extended with the introduction of tip loss correction factors [96, 97]. Recently in 2016 Koh and Ng [98] provided a good comparison of accuracy between three tip loss correction models in tidal turbines. Further developments have resulted in models accounting for separation and other factors; these include dynamic inflow models [99], yaw and tilt models [100] and dynamic stall models [101]. Further descriptions of these can be found in [95].

### Actuator Disc (AD)

The AD model is the most simplified CFD approach that can be used for turbine modelling; it is based on the one dimensional conservation of linear momentum through a control volume. The turbine blades are modelled as a porous disc that extracts energy from the upstream flow. The force exerted on the flow by the blades is distributed over the disc and is a function of the turbine thrust coefficient, found using engineering lookup tables.

Since the development of this simplified model, researchers have constantly improved it. For example, the distribution of the thrust forces can be achieved in numerous ways, from a simple uniform distribution [81, 102] over the rotor swept area to more complex fourth order polynomial approximations [103]. In 2007 and 2008 Jimenez et al. [80, 104] modelled the forces across the disc in a uniform manner, focusing on the spectral coherence of the turbulence within the Nørrekaer Enge II Windfarm. Then in 2014 Simisiroglou et al. [103] compared a uniform and polynomial distribution of thrust forces over the disc area using different turbulence models. The Lillgrund offshore wind farm, off the coast of southern Sweden provided experimental results with which to compare the numerical results. It was concluded that the polynomial distribution of forces more accurately modelled the turbine wake aerodynamics, with a better match to the experimental results.

More advanced methods have used BEM theory to add rotational effects into the flow [81, 105]. These studies represent two examples that have compared the AD method with both rotational and non rotational factors. Wu and Porté-Agel [81] in 2010 showed that in the far wake region (ten rotor diameters downstream [106]) the AD method with rotational effects showed little difference to the AD model without rotational effects. In 2013 Aubrun et al. [105] showed experimentally similar results, that in the far wake region a porous disc closely modelled three rotating blades in the level of turbulent intensity and stream wise velocity.

The AD model does not require a fine mesh resolution around the blades, which makes it computationally cheap, ideal for large domains, containing turbine arrays. In 2012 Gonzalez-Longatt et al. [107] investigated the impact of wake effects on the operation of the wind farms, considering both the short and long term effects on power output. Avila et al. [108] offered a proof of concept study to modelling CFD farms and its ABL on large computing systems. Whilst in 2013 Castellani et al. [109] and in 2015 Creech et al. [110] used the AD model with RANS and LES respectively to model offshore wind farms.

The AD model does not represent finite blades, the simplified representation of the turbine rotor means the model cannot simulate any boundary layer effects on the blades [12], and struggles to capture blade tip vortices and more complex physical phenomenon that occur in the near wake region of a turbine. However it can provide a good representation of the far wake, and is useful for computationally expensive simulations looking at larger flow scales, such as those found in the ABL. The next “step-up” for modelling the turbine is the Actuator Line Method.

### Actuator Line (AL)

The AL method developed by Sorensen et al. [111] modelled the blades as distinct lines as shown in Fig. 3, as opposed to a disc covering the swept area. The loading on each blade can be determined through available airfoil data. Results in [111] and [12] show that there is a distinct difference in the near wake between the AD and AL method. The AL method is able to capture the tip vortices as distinct vortex tubes in the near wake region, as opposed to a sheet in AD simulations.

Studies[12, 112] have compared the AD and AL methods for modelling the wake in turbines. In 2014 Wilson et al. [112] compared the AD, AL and a fully resolved model and compared the numerical results to the NREL 5MW reference turbine. A similar study by Martinez et al. [12] in 2015, summarised the differences between the AD and AL methods and again used the NREL 5MW turbine experimental results [113] as a reference. They used a non turbulent and uniform inflow, with a turbine operating at its maximum energy extraction capabilities. The mean wake profiles for both these methods is almost indistinguishable, however the AL method creates tip and root vortices that are convected downstream. This slight difference in the way the downstream wake is formed results in two different methods of wake breakdown. The AD method results in axi-symmetric Kelvin–Helmholtz instability and roll up, whereas the AL method disrupts this symmetry with its tip and root vortices.

Many studies involving the AD and AL methods do not include the effects of the nacelle within the models, however Lu and Porté-Agel [88] used the AL method and included the effects of a nacelle by modelling it as a solid bullet shaped object, to investigate the effects of the ABL on large wind farms.

Further examples of authors using the AL method include Machefaux et al. [114] who compared the results from computational simulations with experimental results taken from the Tjaereborg wind farm in Denmark, whilst Churchfield et al. [115] simulated the Lillgrund wind farm using the LES and AL methods.

### Actuator Surface (AS)

An advancement on the AL method is the AS approach developed by Shen et al. in 2007 [83]. The blades are represented by points distributed over a surface, representative of the blade shape, shown in Fig. 3. The distribution of forces over the blade was determined using XFOIL [116], a computer program that resolves the viscous boundary layer over a blade. The study revealed that the AS technique more accurately predicted the flow structures at the blade edges and tips.

Many studies have been conducted using the AS method. In 2007 Dobrev et al. [117] offered a Hybrid approach using BEM and the AS model in the CFD code Fluent 6.2 and found this method was suitable up to wind speeds of around 10 m/s. It fails to accurately simulate higher wind velocities because the method does not account for radial forces, which become more prevalent at higher wind speeds. In 2009 Shen et al. [118] used the AS approach to model a vertical axis wind turbine and Sibuet Watters et al. [119] compared numerical results using the AS method with experimental values from the NREL phase VI rotor. Good agreement was found between the two sets of results from high tip speed ratios, or until stall begins to occur. More recently, in 2015, Kim et al. [84] improved the AS model to remove the over prediction of the thrust and power coefficients at the hub and tip areas previously seen in AS methods.

It was found in [117, 118, 120] that although the actuator surface model goes further in accurately modelling some of the flow in the near wake, such as the structure near the blades and the root and tip vortex regions, it fails to model the flow well at high inflow speeds, when the flow is detached from the blades and the three dimensional effects, ignored in BEM theory, become more dominant.

### Fully Resolved Models (FRM)

At the top of the hierarchy is a FRM of the turbine blades. FRMs provide more accurate predictions of the flow around blades and offer engineers more detail about the smaller scale structures and loads on the blades.

A large proportion of the work [82, 92, 121–123] undertaken using FRMs to study rotors in more detail has been carried out by the Risø National Laboratory, Wind Energy Department at the Danish Technical University. They use the EllipSys3D program, which uses a multi block finite volume approach based on the incompressible RANS equations, and makes use of the Message Passing Interface (MPI) library to run simulations in parallel.

A number of the early studies in 2002 and 2003 [82, 121, 124, 125] compared their results with experimental data taken from the comprehensive tests done by the National Renewable Energy Laboratory (NREL), in the US, on the Phase VI turbine [126]. The Phase VI is a two bladed turbine with a 10 m rotor diameter. It was encased in an 80 by 120 foot tunnel and rotated at 72 rpm.

Sorensen et al. [82] ran two configurations, one in free conditions and the other with a surrounding tunnel using a *k*−$\omega$ SST turbulence model. It was found that the model accurately simulated the 3D flow effects on the rotor below stall conditions, however it failed to accurately predict the rotor power at higher wind speeds (>10 m/s) when the flow detached from the blade. Johansen et al. [121] ran similar models using a Detached Eddy Simulation (DES) method, finding that the DES did not improve on the results obtained using the *k*−$\omega$ SST model.

Duque et al. [125] compared the analytical lifting line computer program, CAMRAD II, with OVERFLOW-D, a structured grid RANS solver, on the NREL Phase VI. It was found that OVERFLOW-D predicted the performance of the rotor well in stalled conditions which CAMRAD II failed to predict.

In 2003 Benjanirat and Sankar [124] studied a number of turbulence models and their accuracy at predicting forces and bending moments at the blades, using a 3D Navier Stokes program developed at Georgia Institute of Technology. In 2005 Tongchitpakdee et al. [127] used the same program to study the effects of three turbulence models at four wind speeds and four different yaw angles. Results showed that for highly separated and fully attached flows, the turbulence models used had little effect on the accuracy, however for partially separated flows they determined more sophisticated models and better mesh resolution was required, to produce higher fidelity results.

In 2005 and 2006 Schmitz et al. [15, 128] used CFX V5.6 and V5.7 respectively, which combines RANS equations and the Vortex Panel Method (VPM), in a series of studies. This method combined the ability of the RANS to model 3D effects at the blade tip and root with the VPM’s to model the convection of the vortex sheet downstream. The VPM does not suffer from numerical dissipation of vortical structures that RANS models do [129], however the flow is assumed to be fully attached to the blade.

More recently, in 2011 there were multiple studies using Finite Element Methods (FEMs) to model the incompressible Navier-Stokes equations. Bazilevs et al. [10, 11] have produced a comprehensive methodology for modelling the geometry of the turbine rotor and the aerodynamics associated with it. The study made us of quadratic Non Uniform Rational B-Splines (NURBS) to model the geometry, whilst the incompressible Navier–Stokes equations were solved using a Residual Based Variational Multiscale (RBVMS) method. This methodology has subsequently been used by Hsu et al. [9, 130], considering the capabilities of the method for large computing systems and its future in fluid structure interaction.

Takizawa et al. [131] compared two advances on the basic Deforming Spatial Domain/Stabilised Space Time model (DSD/SST) to study the NREL 5MW offshore baseline wind turbine. The DSD/SST is a FEM adapted to work with moving boundaries and interfaces [132, 133]. They compared the torques on the blades of each method to a reference value [10]. This study highlighted the numerical performance of stabilisation methods used to study loading on a turbine rotor.

In 2016 Zhou et al. [134] used ANSYS Fluent 14.0 to study the effect of different inflow conditions on the NREL phase VI wind turbine. The LES study compared the results from a number of inflow conditions: uniform, vertical linear wind shear and vertical linear wind shear with turbulence present. The conclusions showed that wind shear must be taken account of when studying both the wake and loading on the blades. It introduced cyclic loading onto the rotor not seen in the uniform inflow case, and resulted in a non symmetric, non uniform far wake structure. This is an example of a study modelling the wind resource more accurately; many studies done by the Wind Engineering and Renewable Energy Laboratory (WIRE) in Switzerland have considered the effect that atmospheric stability can have on wind farms, some of which will be discussed in Sect. 5.

#### Rotor–Tower Interaction

The interaction between the tower and rotor is in its infancy with a limited number of studies. The first full 3D analysis of a down-wind turbine configuration was by Zahle et al. [92] in 2009. The upwind turbine has been looked into a greater number of times, with the first study being done by Gomez-Iradi et al. [135] in 2009, and further research undertaken since 2010 including [93, 123, 136].

The downwind configuration of the NREL phase VI turbine was studied by Zahle et al. [92], due to the recent introduction of the overset grid method into EllipSys3D. This led to better techniques to deal with the relative movement between the rotor and the tower/nacelle. The model was able to preserve the tip vortices for approximately 1D downstream before dissipating, which is believed to be a result of mesh resolution. The study further highlighted the tower shedding frequencies dependence on the rotor. The torque and thrust experienced by the blade was determined by whether it passed through a region of positive or negative vorticity shed from the tower.

Li et al. [136] again used an overset grid technique with an incompressible unsteady RANS and DES solver, in CFD-Ship-Iowa V4.5, to study the NREL phase VI at varying wind speeds and blade pitch. This study used a finite difference approach using a blended* k−* $\omega$/*k−* $\epsilon$ SST turbulence model. The domain contained $52.3\times 10^6$ grid points distributed across 2048 processors. Simulations were run by fixing the pitch and varying the wind speed and vice versa, subsequently comparing the power and thrust calculated to experimental results. The CFD simulations predicted the values well, however for more demanding wind speeds, where large separation occurs on the blades, the thrust was over predicted and the power under predicted. The DES caught fluctuations of thrust and power at similar frequencies however, in general, it under predicted the amplitudes.

There have been comprehensive studies concerning the aerodynamics of wind turbines, from the intricate details at the blade tips, obtained through FRMs to the mean wake characteristic present in large arrays obtained using AD/AL methods. Selected papers are summarised in Table 2.

**Table 2:** Summary of papers using fully resolved models

Paper	Test case	Software	Year	CFD model	Turbulence model
[82]	NREL phase VI	EllipSys3D	2002	RANS	$k-\omega \, SST$
[121]	NREL phase VI	EllipSys3D	2002	DES	–
[124]	NREL phase VI	NASCART-GT	2003	RANS	$B-L,S-A,k-\epsilon$
[125]	NREL phase VI	CAMRAD II&	2003	RANS	$B-B$
		OVERFLOW-D			
[15, 128]	NREL phase VI	CFX 5.6 & 5.7	2005, 2006	RANS	$k-\omega , k-\epsilon$
		+VPM			
[137]	Siemens SWT-2.3	CFX 10.0	2007	RANS	$k-\omega \, SST$ Langtry/Menter
[122]	NREL phase VI	EllipSys3D	2007	RANS	$k-\omega \, SST$
[135]	NREL phase VI	–	2009	RANS	–
[92]	NREL phase VI	EllipSys3D	2009	RANS	$k-\omega \,SST$
[123]	MEXICO	EllipSys3D	2011	RANS	$k-\omega \, SST$
[138]	NREL 5MW	–	2011	RBVMS	–
[93]	MEXICO	OpenFOAM	2012	RANS	$k-\omega \,SST$
[134]	NREL phase VI	FLUENT	2016	LES	–

## Wind Farms

Wind turbines are usually grouped together into large arrays called farms. The power output from a wind farm is not as simple as the sum of the rated powers of all the turbines. The leading turbines extract energy from the wind resulting in a wake. This leads to a reduced power output from preceding turbines and higher unsteady loads on the rotor and tower [139]. The velocity deficit between the free stream and wake region is usually negligible after ten rotor diameters, however the turbulence intensity difference can be detected as far as 15 rotor diameters downstream [94]. For a detailed review of wind turbine wake aerodynamics, the reader is directed to Sanderse et al. [14].

There are three main sources of turbulence from a wind turbine. Firstly, atmospheric, this is caused by the roughness of the earth’s surface and the stability of the ABL. Secondly, mechanical, caused by the interaction between the flow and the turbine rotor and tower and finally, the shear layer, caused by the tip vortices breaking down into the main wake body.

Modelling wind farms is key to maximising the energy extracted from the wind and minimising the power losses due to the wake incurred downstream of the leading turbine. As the number of turbines increases, it becomes more computationally expensive to model [108]. A large number of wind farm studies [140, 141] make use of the simplified rotor models described in Sect. 4.

There have been many studies conducted in the area of wind farm aerodynamics, with authors looking at a large combination of factors, from wake analysis [85, 86] and the interaction and effect they have in farms [142], to using these models to study wind farm layouts and more recently the effect large wind farms can have on local climate. They are all interlinked, but two major categories emerge from the literature:

1. Wake analysis
2. Layout optimisation.

### Wake Analysis

Wake analysis involves the study of the wake interaction models and their accuracy and application for multiple turbines under varying conditions, different layouts and inflow parameters. In 2011 Sanderse et al. [14] provided a review of wake aerodynamics for different rotor modelling techniques.

The main issue with analysing a wake model is the variability and uncertainty in collected data. Barthelmie et al. [139] studied multiple different wake models from software used for wind turbine modelling; WAsP, WakeFarm, WindFarmer and NTUA and compared them to observational results taken from Nysted and the Hors Rev wind farms in Denmark. The models were able to capture the wake widths well at 10 m/s however the author concluded that the uncertainty in the models was still too high. All the models made approximations to resolve the near and far wake. NTUA is the most sophisticated model, using RANS equations in combination with the AD method. This is suitable for industrial applications as they are computationally quick and provide good results for mean quantities in the flow field. However models based on 1st principles are required to resolve some of the finer details.

Tabib et al. [141] studied the difference between RANS and LES models in the onshore Bessaker wind farm. The models used the AL method and included the effects of terrain in the study. It was found that the RANS model does not capture the wake dynamics, including the wake decay and interference as well as the LES model.

Many studies have been undertaken using LES [85, 86, 88, 140, 143] in order to better understand and resolve the wake aerodynamics of turbine arrays and the effects large farms can have on the stability of the ABL.

Calaf et al. [85, 86] discovered that if the length of the wind farm is greater than the size of the ABL then the flow through the farm enters into a fully developed regime; in this situation the main transfer of energy comes from the vertical transport of momentum. The study used the AD model to reduce the computational time of the simulations. They studied a range of different farm layouts, turbine loading factors and initial surface roughness values to study the mean statistical data throughout the farm. A key finding was that the vertical fluxes of kinetic energy were of the same order as the power extracted by the turbines. It was further discovered that there was between a 10–15% increase in the scalar fluxes in the ABL due to the higher turbulence, induced by the turbines, and so increased turbulent diffusivity and mixing.

In 2011 Lu et al. [88] used the AL model along with LES to model a large wind farm in a stable atmospheric boundary layer. The study showed how the effects of non-uniform incoming turbulence, the Coriolis force and rotational effects from the blades lead to a skewed spatial structure in the wake with some of the turbulence being driven away from the centre. Clear asymmetric loading were seen on the blades due to the inclusion of high wind shears in the incoming flow. Lu et al. [143] further used the same model to study the effects of a large wind farm on a convective ABL. Both aligned and staggered farms were studied with different streamwise and spanwise spacings. The presence of the wind farm altered the stability of the ABL, by increasing the height of the boundary layer and magnitude of the heat flux from the surface. It is hoped studies such as these provide information that can be used in weather and climate models.

Porté-Agel et al. [140] developed a model using LES and the AD model including the rotational effects of the blades. This was compared to experimental results of a scaled wind farm test carried out in a wind tunnel. This model was then used to compare the lateral wake interaction in both aligned and staggered wind farms. The staggered setup showed much stronger wake interaction than the aligned one.

In 2015 Goit and Meyers [144] published an interesting study where the turbines where used to optimally influence the transport of momentum and energy vertically. Optimising the vertical fluxes led to increased turbulent dissipation within the farm and hence a reduced wake recovery.

Large wind farms can have big impacts on the local and global climate. A number of studies have highlighted the effects they can have on the local climate. In 2004 Baidya Roy [145] and Keith et al. [146] showed that the increased turbulence from the farms leads to increased vertical mixing of momentum and heat, which can have significant effects on the surface temperatures. Baidya Roy et al. [147] showed using a regional atmospheric modelling system [148], that under stable atmospheric conditions, there is a warming effect underneath the farm boundary layer. Wang et al. [149, 150] found results agreeing with [147] and also showed that in offhsore wind farms the opposite occurs, there is increased surface cooling.

Moving forward with generating accurate and usable models, a proof of concept study by Rasheed et al. [151] has provided a methodology for fluid structure interaction modelling of wind turbines, and coupling this to meteorological codes that model the micro and meso scale meteorology. A model such as this could provide accurate power output data for the turbine along with better predictions about the fatigue due to the incoming turbulence.

**Table 3:** Summary of papers simulating farms

Paper	Rotor model	CFD model	Test case	Year
[102]	AD	RANS	Sexberium	2013
[89]	AD + parabolic wake model	RANS	Sexberium	2014
[85, 86]	AD	LES	–	2010, 2011
[88]	AL + BEM	LES	–	2011
[115]	AL + FAST	LES	–	2012
[87, 90, 152, 153]	AD + R	LES	–	2013–2016
[154]	AL	LES	Lillgrund	2013
[110, 143, 155]	AD + BEM	LES	Horns Rev, – , Lillgrund	2015
[156]	AD	LES & WRF	Lillgrund	2015

### Layout Optimisation

In order to maximise the energy output from a wind farm the positioning of individual turbines must be decided carefully. This can be considered as an optimisation problem. Optimisation problems are computationally expensive with 100 s and sometimes 1000 s of solutions considered, and the objective function depends on a range of factors that can all affect each other. Some important variables to consider during the development of the optimisation function include: initial capital costs, wind speed and direction, turbine and farm losses and operation and maintenance costs. Optimising the layout of wind farms is important in maximising the output of energy at the lowest cost, making wind energy more competitive compared to sources such as oil and gas.

In formulating the optimisation problem some assumptions and constraints have to be made. At this moment in time it is not feasible to model turbine arrays using CFD methods, so simplifications are made. A wake model developed by Jensen [157], is one of the most common models used to model the deficit and losses in turbine arrays. It assumes momentum is conserved within the wake stream and the wake expansion is linear and a function of distance from the turbine [158].

Studies have used a wide range of methods to solve the optimisation problem. Some of the first studies used genetic algorithms [159–162] with a discrete computational domain. Other solution strategies have included, Monte Carlo methods [163], mathematical models [164], evolutionary algorithms [165–167], ant colony algorithms [168] and particle swarm optimisation [169, 170]. Software used in industry such as WindPro and WindFarm use heuristic methods to optimise the wind farm layout.

In studies between 1994 and 2007, the domain was discrete, with each grid square representing a set area and possible WT location. However in 2010 Kusiak and Song [165] presented one of the first studies in which the turbine location was represented by coordinates in a continuous circular computational domain. The study assumed a fixed number of turbines with any two being separated by at least four rotor diameters. A Weibull distribution was used to approximate the wind speed at the hub height and an evolutionary algorithm was used to solve the optimisation problem.

Gonzalez et al. [167, 171] produced a comprehensive analysis of the WT cost using net present value as the cost function. The study included initial capital costs, wake decay effects, auxiliary costs and the electrical infrastructure in its cost analysis, whilst also considering different types and sizes of turbines. It used a Weibull distribution and wind rose to model the wind speed and direction respectively.

In 2015 Chen et al. [172] researched the possibility of using turbines with different tower heights. It used a genetic algorithm with two different tower height possibilities, in a 2 by 2 km domain considering only a constant wind speed and direction. For this limited study it was concluded that using turbines with different heights could be beneficial to total power output.

In 2016 Song et al. [23] used the study in [165] and considered 3D optimisation of WT layout. It extended the 2D wake loss model by including the wind profile and geometric height into the analysis. The computational study considered two cases, low and high variability in the wind direction. It was in agreement with [172] and extended this to conclude that for variable winds, different sized turbines positively benefited the power production of the farm in the long term, however this effect gradually decreased as the wind direction became more stable.

Some studies have included the effects of landowner costs into their objective functions [173, 174]. These models add an extra constraint into the problem but are useful when considering wind farm siting.

The linear wake model used in many of the studies described above and the following, [158, 175–178] cannot be used for more complex terrain and non-uniform flow fields. A method developed in [179] known as the virtual particle model has been used by studies [180–182] considering complex terrain. The increased computational demand of this wake model means genetic algorithms cannot be used, as they generate huge populations that must all be resolved. The studies use evolutionary methods that start with an initial layout, then go through cycles of adding, moving and removing turbines to settle on an optimised layout.

Table 3 summarises the papers discussed in this section. It is clear to see a majority of the research undertaken has made use of LES with one of the simplified rotor models to reduce the computational expense that resolving the turbines brings.

## Turbine Mechanics

Turbine mechanics covers a whole range of areas in wind turbines, that essentially involves any of the non aerodynamic factors associated with turbines. Some of these are:

1. Structural analysis of the blades
2. Mechanical components in the hub and nacelle
3. Power control methods

The structural analysis of the blades has been researched intensively since 1993 [183], that began by extending research in the aeronautical industry, in particular helicopter technology, to wind turbine rotor blades. From this a series of methodologies have been produced to model the blades, almost all involving FEMs.

**Fig. 4:**
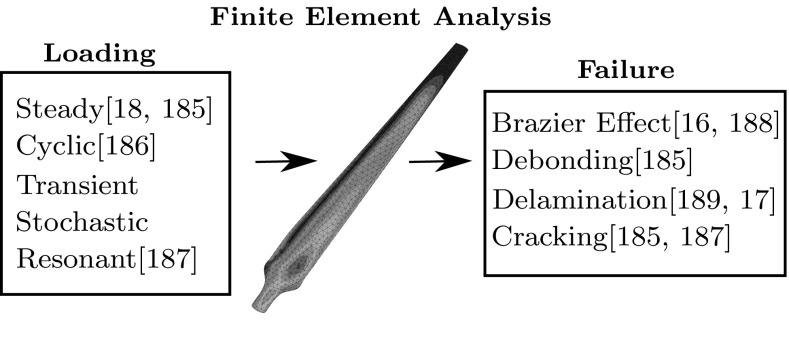
Consideration of the loading and failure mechanisms of a turbine blade using finite element analysis

Turbines are designed to last a minimum of 20 years, which is equivalent to approximately $10^8$ rotations of the blades. The turbine must withstand a range of different loads that can cause considerable damage and fatigue to the structure. Common load types incurred are shown in Fig. 4.

Main sources of loading come from the aerodynamic effects. Turbines must be able to withstand the effects of high winds and stochastic loads such as turbulence, in a range of different positions. Transient loads are generated during start-up, braking and yawing of the system, and can lead to large stressing of joints and bearings within the system.

One of the first models developed to study the turbine as a single system was the Linearized Hinge-Spring Blade rotor model, developed in 1987 by Eggleston and Stoddard [189], which is a simple model for the analysis of the deformation incurred by the turbine. It has an analytical solution that accounts for steady and cyclic loads, modelling each blade as a rigid body attached to a rigid hub through a series of springs and hinges. The model is a first order model that fails to predict higher frequency oscillations throughout the structure, so more developed models have been produced to resolve the finer details of turbine dynamics.

FEM has become increasingly popular to model the static and dynamic deformation of blades and structures in wind turbines. More recent studies using static analysis methods such as that by Jensen et al. [16] considered the geometrically non linear structural deformation of a blade. It took into account elastic effects such as buckling using a combination of solid and shell elements, however it neglected any damage criterion such as crack propagation or delamination. In the following year, Yang et al. [184] compared a full scale experiment to numerical results analysing the structural collapse of a blade. It included geometric non linear effects using shell elements. In 2015 Lee et al. [18] simulated the static analysis of the wind turbine blade root.

A transient analysis is often more useful due to the information it provides. Blades are increasingly failing due to vibratory and fatigue loads induced through cyclic loadings, which transient models can account for. In 2012 Lee et al. [79] used a flexible multi-body dynamic solver to analyse the aeroelastic response of a turbine blade. In 2014 Yu and Kwon [190] used FEM with non linear coupled flap-lag torsion beam theory, in combination with a CFD model, to again study the aeroelastic response of full wind turbine configuration. Gebhardt et al. [191, 192] used a segregated formulation of the structural equations, with an aero elastic model to predict the elastic response of the blade.

### Failure Criterion

The loading on a wind turbine can be categorised into ultimate loads or fatigue loads. Ultimate loads are the maximum forces that can be withstood by the structure and its components, where as fatigue loads consider the ability of the structure to withstand cyclic loading. The most obvious periodic/cyclic load is the stress at the blade root caused by gravity and rotation of the rotor.

The blade is considered the most important part of the wind turbine as its structural integrity and ability to convert kinetic energy of the wind to mechanical energy, is paramount to efficient and cost effective turbines. As such there have been a number of experimental and numerical studies considering both the ultimate [16, 18] and fatigue [193, 194] failure of the blades. The cause of failure in turbine blades has an inherent link with research areas involved in composite laminates [17, 188] and analysis of thin structures [184].

Modern blades are made of polymer-matrix composites, and undergo approximately $10^8$ cycles/rotations in their 20 year lifetime. With such a large number of cycles even the smallest periodic loads can dramatically fatigue the structure [195].

A number of studies have aimed to pinpoint the main failure system in turbine blades, Fig. 4 summarises the failure mechanisms that will be described below.

Jensen et al. [16, 187] determined that the Brazier effect [196] was the main cause of failure. The Brazier effect arises due to the bending of thin walled hollow structures, such as the shear web within the blade, and causes a crushing pressure on it. They used solid and shell elements to analyse the elastic, geometrically non-linear structural behaviour of the structure using FEM.

Overgaard et al. [17, 188] considered the primary failure mechanism to come from delamination within the composite layup which led to buckling. They produced an FEM model capable of modelling the propagation of inter-laminar failure.

Yang et al. [184] discovered that under flapwise loading, stress concentrations around defects within the material cause an initial fracture which, in combination with delamination and debonding of the aerodynamic shell, propagates to cause complete structural collapse.

Chou et al. [186] analysed the failure mode of blades damaged in a typhoon that hit the Changhua Costal Industrial Park in Taichung. An finite element model was produced based on the criteria found during the field analysis of the blades, which was then used to simulate both a static and dynamic load on the blades. It was found that the resonance of the blade over long term can lead to the formulation of cracks and delamination. This would lead to progressive damage within the blades and subsequently under high wind conditions failure.

In 2015 Lee et al. [18] undertook an experimental study, discovering that the onset of failure began from delamination at the blade root. Subsequently an FEM model of the blades was produced in Abaqus using a combination of shell and solid elements. A static analysis was undertaken which showed that conventional methods of modelling the root as a hollow circular cylinder does not represent the real stress distribution. It emphasised the difference in loading across the bolts used to join the blade to the hub.

The above studies all use finite element models to clarify and obtain more detail about the initial causes of failure in turbine blades. Along with using previously existing finite elements to model wind turbines, some studies [197, 198] have focused on the development of finite elements specifically to model turbine blades. There has been a number of purely experimental tests [199, 200] that do not fall under the scope of this review, but are useful for the analysis of blade failure mechanisms.

Fatigue analysis has been used in [185, 193, 194, 201] to improve the approximation of lifespans. It is usually modelled using empirical formulae developed by Spera in [202] in conjunction with S–N linear damage equation, Miners law and Goodman diagrams. The basics of fatigue in turbines is described in [195].

Kong et al. [185, 194] undertook a full analysis on a 750 kW blade made of E-glass and epoxy, using a full scale experimental test to validate the FEM model developed. The model included a wide range of loading from aerodynamic and mechanical loads to effects of ice and extreme conditions. The study showed good agreement between the experimental and numerical model.

Marin et al. [201] used S–N curves along with miners rule to identify the fatigue mechanics that were predicted to have caused failure in a 300*kW* turbine blade. The analysis showed that the onset of fatigue caused a superficial crack to be formed which propagated, causing further cracking and delamination, eventually leading to failure of the blade.

### Turbine Design

The design of blades is critical in the development of longer lasting turbines with lower maintenance costs. With the size of blades growing there has been a trend towards composite materials such as glass fibre reinforced polymers to lower the weight and improve stiffness. In 2015 Chenouri et al. [21] produced a comprehensive review of optimisation methods and techniques used towards improving wind turbine performance.

The design of blades can be split into two categories, aerodynamic design and structural design. Aerodynamic design involves the geometric shape with respect to the inflow conditions: wind speed, wind shear, rated power and tower height to name a few. The structural design of the blade involves the optimisation of the blade, with outer geometric constraints from the aerodynamic design, to reduce factors such as cost, stress and fatigue in the blades.

The objective functions have focused on a range of factors from minimising the blade mass as in [203, 204] to minimising the cost of energy as in [205, 206].

#### Aerodynamic Design

In order to design efficient, light and stiff blades there have been many models that take the form of optimisation problems, with inputs from the aerodynamic and structural factors and outputs of a given blade design.

The objective function of the problems has most commonly taken two forms that focus on the performance of :

1. Aerodynamics [207–212]
  Maximise the lift
  Maximise the lift to drag ratio (L/D)
2. Economics [205, 206, 213]
  Minimise the cost of energy (COE)
  Maximise the annual energy production (AEP)

In 1999 Fuglsang and Madsen [205], aimed to minimise the COE, which is based on a number of factors: the cost of manufacture and erection, the structural fatigue and extreme loads imparted on the turbine, the AEP and finally the noise considerations. The optimisation algorithm consisted of a multi objective model using sequential linear programming; the results were tested using BEM theory with added tip loss corrections included. The methodology reduced the cost of energy in a 1.5 MW stall regulated turbine by 3.5%.

Benini and Toffolo [206] also used BEM theory with tip loss corrections to test their solutions generated using a multi objective evolutionary algorithm aiming to minimise the COE and maximise the AEP. These are two conflicting issues and so a pareto-optimal design space is found as opposed to a single solution. The design variables included the hub/tip ratio, chord distribution, twist and tip speed ratio.

Xudong et al. [213] used similar design variables and flow model as Benini and Toffolo, but included a dynamic structural model to analyse flap-wise and edgewise deflections. The framework was used to optimise three current rotors, Tjoereborg 2 MW, MEXICO 25 kW and NREL 5 MW, by altering the chord and twist angle along the blade radius. The study only considered the cost of the rotor itself but showed a reduction in COE by 1.1, 3.4 and 2.6% respectively.

A more recent study by Ribeiro et al. [207] used artificial neural network methods along with a genetic algorithm to maximise the L/D ratio. The analysis of the solutions used the incompressible Navier–Stokes equations and the Spalart-Allmaras turbulence model in Fluent. A pareto front was found, with one solution offering the lowest coefficient of drag and another the highest coefficient of lift.

Other authors have used unique objective functions, with Jeong et al. [214] aiming to minimise the fluctuating flap wise bending moment of the blade in turbulent wind and Lee et al. [215] minimising the noise produced by the airfoil of a 10 kW turbine.

#### Structural Design

The structural design of a blade is heavily linked with the fatigue and failure modes described in the previous section. With the concern about the blade weight and stiffness, there was a movement away from metals towards composite materials. There had been a number of studies [216–218] concerning the optimisation of composite laminates in structures. There have also been comprehensive studies into optimisation techniques [219] used in physical sciences. A combination of these two research areas led to the optimisation of the structural design of wind turbine blades.

Jureczko et al. [220] used a modified genetic algorithm in a multi-criterion optimisation model to minimise vibration, maximise power output, minimise blade cost and fulfil any strength requirements of the blade. This was done using the following criteria: the shell and web thickness and the number and position of stiffening ribs. The models were tested using the FEM in Ansys to ensure strength and displacement requirements of the blade were met.

A similar study was conducted by Chen et al. [22]. A BEM model was used to predict the aerodynamic forces which are applied to the proposed design and modelled using the FEM method. A particle swarm method was used to alter the spar cap position and thickness to minimise the mass of the blade.

Lund and Stegmann [221] used a gradient based optimisation technique to design the blade to maximise the stiffness. The composite layup of the main spar is altered using a discrete material optimisation approach and the subsequent model stiffness is calculated using a weighted sum of the candidate materials and linear buckling analysis in the FEM. This optimisation methodology was similarly used in [222] and [223] to optimise the fibre direction in spar caps, with the former assuming linear elastic behaviour and the latter using non linear buckling analysis to model the material.

Monroy Aceves et al. [224] have produced a methodology for material selection and design, that combines properties of the material with results of non-linear finite element analysis of the model, based on aerodynamic forces. The solutions are then reduced down based on specific selection criteria to choose an exact design.

The optimisation of blade shape and structure will become increasingly important as the blades grow in size and flexibility.

## Fluid–Structure Interaction

**Fig. 5:**
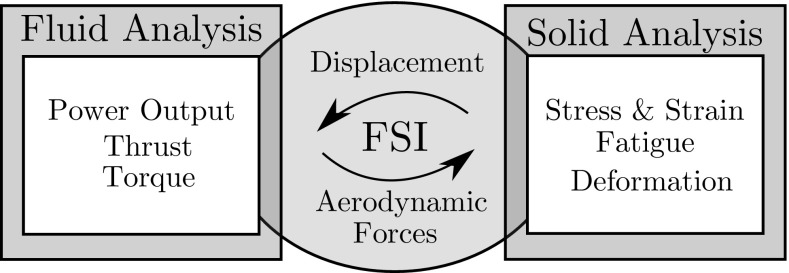
Basic transfer of data in FSI simulations with important outputs from the fluid and solid analysis

Fluid structure interaction (FSI) involves coupling the fluid and structural solutions each time step to produce a model capable of including the effects of the solid on the fluid and vice versa. Essentially, the force exerted by the fluid on the solid causes it to deform, which in turn alters the flow field, see Fig. 5.

FSI has been present in the wind industry under the name of aero-elasticity [190, 225, 226] for some time. Aero-elasticity involves coupling a flow solver to a structural model, to predict the dynamic response of the structure to a range of loads. A series of programs are available that use BEM theory with simple one dimensional structural models [227, 228].

However, with recent improvements to performance and capabilities to both software and hardware in the high performance computing industry, there has been a trend towards full 3D analysis of both the structural and aerodynamic analysis of entire turbines [9, 10, 229].

The main body of work has been carried out by the Department of Structural Engineering at the University of California. The studies have been built upon the development of Iso-Geometric Analysis (IGA) by Hughes et al. in 2005 [230]. The development of this methodology led to the first comprehensive study by Bazilevs et al. [10, 11], as a method to model aerodynamics in turbines. The model has been used to run FSI simulations, using a combination of Kirchoff–Love shell theory and the bending strip method, with only displacement degrees of freedom to model the structure.

The domain was reduced to one blade, a 120° cut. The paper showed the twist angle of the flexible blades undergoes high frequency oscillations, attributed to vortex shedding off the trailing edge and turbulence effects.

This led to similar low magnitude, high frequency oscillations in the torque developed on the blade. In 2012 he proposed a new framework to model FSI problems [231] using T-splines to model the structure and NURBS to model the fluid non matching grid at the interface. It performed well in early simulations, showing no drop in accuracy compared to matching NURBS–NURBS fluid structure grid. These models did not include the effects of a tower or nacelle.

Hsu et al. [9] simulated a full downwind turbine using an FSI model. Their model combined an ALE-VMS finite element fluid domain with a structural domain, discretised using NURBS based iso-geometric analysis. The entire domain consisted of 1,440,425 nodes and 4,828,692 elements. These simulations showed the presence of the tower caused a 10–12% drop in torque generated by a blade as it rotated. It also captured the non symmetric loading on the blades which can cause irregular loading on the hub. These findings are important in studying fatigue and expected life span of turbines.

Yu and Kwon from the Department of Aerospace Engineering at the Korea Advanced Institute of Science and Technology [190], produced a loosely coupled fluid structure interaction solver that was used to model the NREL 5 MW turbine. It modelled the blade using non-linear Euler–Bernoulli beam theory, with lead-lag bending, flapwise bending and deformations due to torque all included in the model. They discovered similar results to [9] in that there was a drop in blade loading as it passed the tower and the interference with the tower caused both loading and displacement oscillations. The tower also caused high frequency vibrations that occurred at the blade root.

Most recently two studies have been conducted that again build on the IGA framework [232, 233], that have focused on developing more accurate methods to model the problem, from new mesh moving techniques in the former to a new method to smoothly connect fluid sub domains in the latter.

Some 2D studies have been conducted looking at the effects of morphing blades on the loads generated [234, 235]. It was concluded that morphing blades performed better when the wind speed was under optimum value but worse when over it. Given the operating point of the blade can be controlled in the design process it was suggested that morphing blades offer a better option than rigid blades when it comes to performance over a wide range of loadings. This 2D model has been improved to a full 3D model in [236].

Full 3D analysis, combining the fluid and structure offers more realistic and physical results that can be utilised to design and build more energy efficient turbines.

## State of the Art

Over the past 15–20 years, wind energy has grown in both maturity and efficiency to a point where it is almost competitive with traditional forms of energy, such as coal and gas. Governments have made extensive efforts to encourage investment in renewable technologies, specifically onshore and offshore wind energy devices, so that they can play a major role in the energy market. The trend over the past 15 years has been towards taller and larger turbines, which are more cost effective than their smaller counterparts. However, the linear and simplified approximations originally developed to model the physics no longer provide the fidelity and detail required by engineers. There is a requirement for models that better simulate both the fluid dynamics and structural mechanics of the problem.

This review has revealed the state of the art in the research being undertaken in the area of “Wind Energy” and “Computer Simulation”. The review has covered a wide range of topics, from atmospheric flow modelling to blade design optimisation.

The wind resource was the first topic covered. In order to generate more reliable and physical models it is important to correctly simulate the input to a wind turbine or farm. The state of the art in boundary layer modelling, combines the mesoscale atmospheric models that capture effects down to around 1 km with large eddy simulations that can capture the finer details to approximately 50 m [76, 77]. The Weather Research Forecasting Model (WRF) is one mesoscale model used, it captures the atmospheric dynamics and can be run across multiple cores in parallel environments. However for more specific modelling of complex terrains, studies have found that RANS simulations can more accurately predict the flow, when compared with experimental results [74].

It has been clear the effect that available computing power has had on the development of rotor models and the CFD approaches used. When modelling turbine rotors and blades, fully resolved geometries combined with RANS simulations using two equation turbulence models, currently dominate the field, see Table 2. They provide results that most accurately match the extensive experimental data available, for a range of turbines. The computational requirements needed to resolve the near wall boundary layer using LES models is high, however in 2016 Zhou et al. [134] performed an LES study using a fully resolved model of the NREL phase VI turbine. This represents a important step towards higher fidelity CFD simulations.

Modelling and simulation involving wind farms, has made use of the recent developments in wind resource modelling techniques to simulate the complex dynamic flows that occur at turbine rotor heights. The farms are modelled using a range of techniques but, the state of the art lies in LES simulations using AD and AL models, see Table 3. These types of models have been used for both wake analysis [86, 88, 140] and layout optimisation [23, 165], in large turbine arrays. These models provide important information about how turbines interact with each other when arranged in a large array.

Research involving the structural mechanics in wind turbine blades has involved research into the failure mechanisms [17, 186, 188]. The FEM is used in a large proportion of the studies reviewed, utilising a range of different finite elements to model the external and internal structure of blades. Optimisation studies into blade structure and shape use the finite element models to validate the final solutions.

Figure 1 represents the division between the scales of modelling and simulation in the wind industry. From 2011, studies [9, 10] begun to couple fully resolved CFD simulations with full 3D geometric representations of the structure in FSI simulations. This represents a large step in coupling the physics of the flow and structure. The state of the art in FSI simulations currently lies in the use of IGA, with studies gone as far as modelling the full turbine [9].

The wind industry faces a range of challenges as the size and number of turbines increases. It is clear that the software used to model and simulate the associated issues will be essential in improving the efficiency and reducing the cost of energy, so wind energy can play a larger role in the energy market.

A large number of models exist to simulate the different scales and physics involved in the wind industry. They will continue to play an important role in the development process, however a clear trend towards models encompassing a range of physics and scales can be seen from the literature. Examples include linking mesoscale numerical weather prediction models to CFD codes in order to improve estimations about incoming wind profiles and ultimately power output from farms. FSI models have been developed to more accurately model the interaction between the turbine and fluid, in order to improve the design of the blades. These models have represented a key step towards full first principal simulations involving multi physics.

The linking and coupling of both scales and physics is possible due to the growing availability of computer resources, HPC systems and cloud computing, to researchers and companies. The opportunities to develop more flexible and scalable software, to improve the fidelity and detail of models, are increasing.

The author believes that with the ever growing computing power available to researchers and the trends towards coupling through scales and disciplines that the “Digital Wind Farm” is a possibility. It strives to encompass and link the individual simulation methodologies currently being undertaken, from mesoscale wind profiles down to damage and fracture models of the structure.

In this era of “Big Data” the amount of information that is obtained from currently existing wind turbines and farms is huge. This data can be integrated into models to add a further level of realism to the simulations. Integration of data into simulations offers many possibilities. The use of LiDAR to measure incoming wind speeds and directions can be used to run pre-emptive/real time simulations to predict power output. The use of sensors on turbine components can be used to provide simulations with data concerning material variability over its lifetime, to better predict lifespan and failure mechanisms.

The gap between the models developed and the industrial solutions produced is slowly reducing. As these models improve and the level of information and detail they provide increases, the wind industry should see more durable efficient turbines being developed. This will result in the wind industry being capable of competing with non-renewable sources and playing a major role in the global energy market.
